# Comparison of oliceridine and sufentanil in patient - controlled intravenous analgesia for post - thoracoscopic nausea and vomiting: a prospective, double - blind, randomized controlled trial

**DOI:** 10.3389/fphar.2025.1576154

**Published:** 2025-09-26

**Authors:** Yuxiang Meng, Sumin Yuan, Hengrui Zhang, Zijie Ling, Chenyang Shi, Yang Niu, Li Zhang, Zhibiao Xu, Yujun Liu, Kang Zhou, Su Liu, Linlin Zhao

**Affiliations:** Department of AnesCesiology, Affiliated Hospital of Xuzhou Medical University, Xuzhou, China

**Keywords:** opioids, oliceridine, thoracoscopic surgery, nausea and vomiting, postoperative pain management, analgesic efficacy

## Abstract

**Objective:**

To compare oliceridine and sufentanil in patient-controlled intravenous analgesia (PCIA) for reducing postoperative nausea and vomiting (PONV) in thoracoscopic surgery patients.

**Methods:**

A prospective, double-blind, randomized controlled trial enrolled 130 patients at the Affiliated Hospital of Xuzhou Medical University from December 2024 to February 2025. Patients were randomly assigned to oliceridine (Group O, n = 65) or sufentanil (Group S, n = 65) PCIA groups. General anesthesia combined with nerve block anesthesia was used intraoperatively, followed by PCIA post-surgery. The primary outcome was PONV incidence within 48 h. Secondary outcomes included nausea and vomiting scores, pain scores, rescue analgesia and antiemetic use, recovery indicators, and adverse reactions.

**Results:**

Baseline and intraoperative characteristics were similar between groups. The primary outcome (48-h PONV incidence) was significantly lower in Group O (32.3% [21/65]) than Group S (50.8% [33/65]; P = 0.033; OR = 0.46, 95% CI [0.23–0.94]), especially within 24 h postoperatively. Moderate-to-severe PONV was also less frequent in Group O (18.5% vs. 38.5%, P = 0.012; OR = 0.36, 95% CI [0.16–0.81]). Early postoperative pain scores were similar, but Group S had higher Visual Analogue Scale (VAS) scores after 12 h (P < 0.05), though the absolute differences were small (e.g., median resting VAS of 0 vs. 1 at 48 h). Rescue analgesic demand and PCIA use showed no significant difference. Group O had significantly higher Quality of Recovery-15 (QoR-15) scores (median difference at 24 h: 5.0 [95% CI 1.2–8.8]; P < 0.05) and significantly lower Athens Insomnia Scale scores (median difference at 24 h: 1.0 [95% CI -2.3 to −0.5]; P < 0.05) compared to Group S. Adverse reaction rates, including dizziness, nightmares, hallucinations, respiratory depression, dry mouth, allergy, and bradycardia, were similar between groups.

**Conclusion:**

In high-PONV-risk thoracoscopic surgery, oliceridine-based PCIA significantly reduced PONV incidence compared to sufentanil (32.3% vs. 50.8%, P = 0.033), while also demonstrating superior recovery quality (QoR-15) and sleep outcomes (AIS). This establishes oliceridine as a procedure-specific analgesic option for enhancing recovery beyond conventional opioid-sparing effects.

## Introduction

In modern surgical procedures, effective pain management and control of postoperative adverse reactions are crucial aspects for ensuring patient recovery. Moderate-to-severe pain after surgery is associated with prolonged hospital stay, readmission, patient dissatisfaction, development of chronic pain, decreased quality of life, and increased costs (Apfelbaum et al., 2003; Wang et al., 2017). Opioids, commonly used drugs for postoperative analgesia, play an important role in relieving pain, but they are also accompanied by a series of adverse reactions, such as nausea, vomiting and respiratory depression (Sun et al., 2023; Paul et al., 2021; Shafi et al., 2018). PONV, which refers to nausea and vomiting occurring in the postoperative period, is the most common symptom after surgery in patients and is affected by various factors, including the type of surgery, the duration of surgery, anesthetic drugs and methods, and preoperative anxiety (Apfel et al., 2012; Öbrink et al., 2015). According to the Apfel scoring system, female sex, non-smoking, a history of previous PONV or motion sickness, and the use of opioids are known risk factors (Apfel et al., 1999). Sufentanil is a potent opioid analgesic widely used in clinical practice. However, the occurrence of its adverse reactions limits its further application (Wang et al., 2023). Oliceridine is a newly developed opioid that was approved by the Food and Drug Administration (FDA) in the United States in 2020 for intravenous injection to treat acute pain requiring opioids (Food and Drug Administration, 2020). As a novel G-protein-biased µ-opioid receptor agonist, Oliceridine selectively acts on the G-protein signaling pathway and has a relatively low potency in recruiting β-arrestin. Theoretically, it can reduce the incidence of common adverse reactions of traditional opioids (DeWire et al., 2013; Ni et al., 2024). While prior studies have confirmed oliceridine’s reduced gastrointestinal effects versus morphine in mixed surgical cohorts (Bergese et al., 2019; Biskupiak et al., 2024), its efficacy in high-PONV-risk procedures—specifically thoracoscopic surgery with baseline PONV rates >40% (An et al., 2021)—and head-to-head comparison against sufentanil (a preferred agent in enhanced recovery protocols) remains unaddressed. This trial uniquely evaluates whether oliceridine’s G-protein bias translates to clinically meaningful advantages over a potent contemporary opioid in a targeted, high-risk population. Thoracoscopic surgery was chosen as the target population because of its inherently high baseline PONV incidence (>40%), synergistic risk factors (e.g., visceral traction, diaphragmatic irritation), and alignment with opioid-sparing Enhanced Recovery After Surgery (ERAS) protocols where gastrointestinal tolerability limits analgesic optimization.

## Materials and methods

This study was a prospective, double - blind, randomized controlled trial. The study was approved by the Ethics Committee of the Affiliated Hospital of Xuzhou Medical University (Approval No.: XYFY2024 - KL591 - 01) and registered at the Chinese Clinical Trial Registry (ChiCTR2400094068). Informed consent was obtained and signed by each patient.

Patients who underwent elective thoracoscopic surgery under general anesthesia at the Affiliated Hospital of Xuzhou Medical University from December 2024 to February 2025 were selected. The inclusion criteria were as follows: age 18–65 years, American Society of Anesthesiologists (ASA) physical status classification I-III, and body mass index (BMI) 18–30 kg/m^2^. Exclusion criteria included: allergy to any drugs used in this trial and contraindications, presence of severe underlying diseases (such as cardiovascular and cerebrovascular diseases, respiratory diseases, or abnormal liver and kidney functions), history of angina pectoris or myocardial infarction within the past 6 months, poorly controlled or untreated hypertension (resting systolic/diastolic blood pressure >180/100 mmHg), long - term use of sedative - hypnotic analgesic drugs, third - degree atrioventricular block, inability to communicate normally (hearing impairment, language comprehension disorder, mental illness, etc.). Patients who could not complete the follow - up, those whose thoracoscopic surgery was converted to open surgery, or those with a surgery duration of more than 3 h (excluded due to the potential for significantly increased tissue trauma, prolonged anesthetic exposure, and heightened inflammatory response—all established independent risk factors for PONV that could confound the primary outcome comparison), those who developed severe hemodynamic disorders or other life - threatening complications, and those who actively requested to withdraw from the trial during the trial process were excluded.

Participants were randomly assigned to either the sufentanil PCIA group (Group S) or the oliceridine PCIA group (Group O) using block randomization with a computer-generated random sequence (created by an independent statistician). Allocation concealment was ensured using sequentially numbered, opaque, sealed envelopes. Upon enrollment, a non-involved anesthesiology nurse opened the next sequential envelope to assign patients to Group O or S. This double-blind design ensured patients, surgeons, and data collectors/analysts remained blinded throughout the trial.

Patients were routinely fasted from food and fluids before surgery. All patients underwent general anesthesia combined with a preoperative ultrasound-guided serratus anterior plane block. The block was performed by an attending anesthesiologist at the level of the 5 th rib in the midaxillary line. Under real-time ultrasound guidance, 20 mL of 0.375% ropivacaine was injected superficial to the serratus anterior muscle, with confirmation of adequate hydrodissection and spread within the fascial plane. After the patients were transferred to the operating room, non - invasive blood pressure (NIBP), electrocardiogram (ECG), and oxygen saturation (SpO_2_) were routinely monitored. A peripheral venous access was established, and radial artery puncture was performed under local anesthesia. Anesthesia induction: intravenous injection of midazolam 0.05 mg/kg, etomidate 0.3 mg/kg, sufentanil 0.5 μg/kg, and rocuronium 0.8 mg/kg. Once the bispectral index (BIS) dropped below 60, muscle relaxation was fully effective, and a double - lumen endobronchial tube was inserted orally under the guidance of a laryngoscope. Proper positioning of the double lumen endotracheal tube was confirmed with fibreoptic bronchoscopy. Mechanical ventilation was then established, and the respiratory parameters were adjusted to maintain the end-tidal carbon dioxide partial pressure at 35–45 mmHg. Anesthesia maintenance: remifentanil infusion was titrated between 0.1–0.3 μg/kg/min based on real-time hemodynamic responses (e.g., MAP and HR fluctuations exceeding ±20% from baseline) and nociceptive stimuli (e.g., surgical incision or retraction),inhalation of 1% sevoflurane, continuous intravenous infusion of propofol 2–6 mg/kg/h, and maintaining BIS between 40–60.

The fluctuation range of intraoperative arterial blood pressure did not exceed ±20% of the preoperative baseline value. Rocuronium 0.3 mg/kg was intermittently added during the operation. Tropisetron 2 mg was intravenously injected during anesthesia induction to prevent nausea and vomiting (Tropisetron is a commonly used 5-HT_3_ receptor antagonist in China for PONV prophylaxis. Although its metabolism is influenced by CYP2D6 polymorphism, clinical studies and practice guidelines support its efficacy in Asian populations, including Chinese patients (Ho and Gan, 2006)). In both groups, the inhalation anesthetics were stopped 30 min before the end of the operation, and flurbiprofen axetil 50 mg was intravenously injected 20 min before the end of the operation. PCIA was initiated immediately after skin closure in the operating room. After the operation, the patients were transferred to the post-anesthesia care unit (PACU). When the extubation criteria were met, the double - lumen endobronchial tube was removed, and the patients were transferred back to the ward when the Aldrete score was ≥9.

In the sufentanil group: The PCIA solution was prepared with sufentanil (2 μg/kg) and tropisetron (6 mg), diluted with normal saline to a total volume of 100 mL. The parameters were set at a continuous background infusion of 2 mL/h, a bolus dose of 0.5 mL, and a lock-out time of 15 min. This delivered a background infusion of sufentanil at 0.04 μg/kg/h and a bolus dose of 0.01 μg/kg.

In the oliceridine group: The PCIA solution was prepared with oliceridine (0.4 mg/kg) and tropisetron (6 mg), diluted with normal saline to a total volume of 100 mL. The parameters were set at a continuous background infusion of 2 mL/h, a bolus dose of 0.5 mL, and a lock-out time of 15 min. This delivered a background infusion of oliceridine at 8 μg/kg/h and a bolus dose of 2 μg/kg.

The dose selection was determined based on previous clinical studies and pharmacological equivalence analysis. Based on the data from APOLLO - 1 and APOLLO - 2 trials, the analgesic potency of oliceridine is approximately 5 times that of morphine (Viscusi et al., 2019; Singla et al., 2019), and sufentanil is 1,000 times as potent (Paul et al., 2021). According to the conversion of equivalent analgesic doses, 0.4 mg/kg oliceridine is equivalent to 2.0 mg/kg morphine, and the calculated equivalent dose of sufentanil is 2 μg/kg, indicating that the analgesic strength of the two is comparable.

Post - operation, the Visual Analogue Scale (VAS) was used to evaluate the pain degree of patients. The VAS score ranges from 0 to 10 points, with 0 point indicating no pain and 10 points indicating unbearable pain. 1 - 3 points represent mild pain, 4 - 6 points represent moderate pain, and 7–10 points represent severe pain. A VAS score of ≥4 points was defined as moderate - to - severe pain. The VAS was also used to evaluate the postoperative nausea and vomiting score of patients (0–10 points), and a VAS score of ≥4 points was defined as moderate - to - severe PONV. If the score on the pain at rest numerical rating scale after surgery was ≥4 points or the score on the dynamic pain numerical rating scale was ≥7 points, and the effect of the analgesic pump was not satisfactory, 100 mg of Bucinnazine Hydrochloride was intramuscularly injected for rescue analgesia. If obvious nausea or vomiting occurred, 10 mg of Metoclopramide was intravenously injected for rescue antiemesis, and the analgesic pump was temporarily turned off if necessary. The pump was resumed once vomiting subsided and the patient’s condition stabilized. During postoperative analgesia, when the drugs in the analgesic pump were exhausted, anesthetic nurses re - prepared the same formula of analgesics to continue analgesia until the use reached 48 h.

The primary observation index of this study was the incidence of PONV within 48 h after surgery in patients, which was defined as any episode of nausea or vomiting. The resting and movement VAS pain scores and VAS nausea and vomiting scores at the time of extubation, 30 min, 2 h, 6 h, 12 h, 24 h, and 48 h after extubation were recorded. The number of patients who required rescue analgesia and rescue anti - emesis within 0–24 h and 24–48 h, the number of patients with moderate - to - severe nausea and vomiting, and the number of patients who pressed the analgesic pump were recorded. The time of the first food intake, the first ambulation, the removal of the thoracic drainage tube, the length of stay in the PACU, and the length of postoperative hospital stay were recorded. The Quality of Recovery-15 (QoR-15) score, the Athens Insomnia Scale (AIS) score, the Bruggrmann Comfort Scale (BCS), and the Ramsay Sedation Score were performed at 24 and 48 h after surgery respectively. Safety evaluation: The occurrence of adverse reactions such as nausea and vomiting, dizziness, and respiratory depression within 48 h after surgery was recorded. The occurrence of hypoxemia (SpO_2_%) was collected by accessing the postoperative nursing record sheets.

The sample size was calculated based on the results of our pre-experiment. In the pre-trial, the incidence of PONV in Group O was 30% and that in Group S was 60%. The sample size was estimated using PASS 15 software. With a power of 0.9 and a significance level of 0.05, the calculated sample size was 106 cases. Considering a 20% dropout rate, a total of 134 patients were finally planned to be included, with 67 patients in each group.

Statistical analyses were performed using SPSS 26.0 software. Normality of continuous variables was assessed with the Shapiro-Wilk test, with p > 0.05 indicating normal distribution. Normally distributed data were expressed as mean ± standard deviation (SD) and compared using independent-samples t-test; non-normally distributed data were expressed as median [interquartile range, IQR] and analyzed with the Mann-Whitney U test. Categorical variables were reported as frequencies/percentages and analyzed with Pearson’s χ^2^test or Fisher’s exact test. A two-sided P < 0.05 was considered statistically significant.

For effect size estimation: Continuous data: Median differences and 95% confidence interval (CI) were calculated via the Hodges-Lehmann method. Categorical data: Odds ratio (OR) with 95% CI were derived from logistic regression models; an OR <1 indicates a lower event risk in the oliceridine group.

For each subgroup, the incidence of PONV in the oliceridine group (Group O) and sufentanil group (Group S) was calculated, and the OR with 95% CI was used to quantify the effect size of oliceridine relative to sufentanil in reducing PONV. Subgroup Analyses: Interaction effects were tested using multivariable logistic regression (treatment × subgroup term). Specifically, a multivariable logistic regression model incorporating the main effects of intervention, subgroup factor, and their interaction term was used to test for interaction. A two-sided P value < 0.05 for the interaction term was considered statistically significant, indicating that the intervention effect differed by subgroup. All subgroup analyses were conducted using SPSS 26.0 software, and forest plots were generated to visually present the OR, 95% CIs, and interaction P values for each subgroup.

Logistic regression was used to calculate OR and 95% CI for primary outcomes (e.g., PONV incidence).

## Results

**FIGURE 1:**
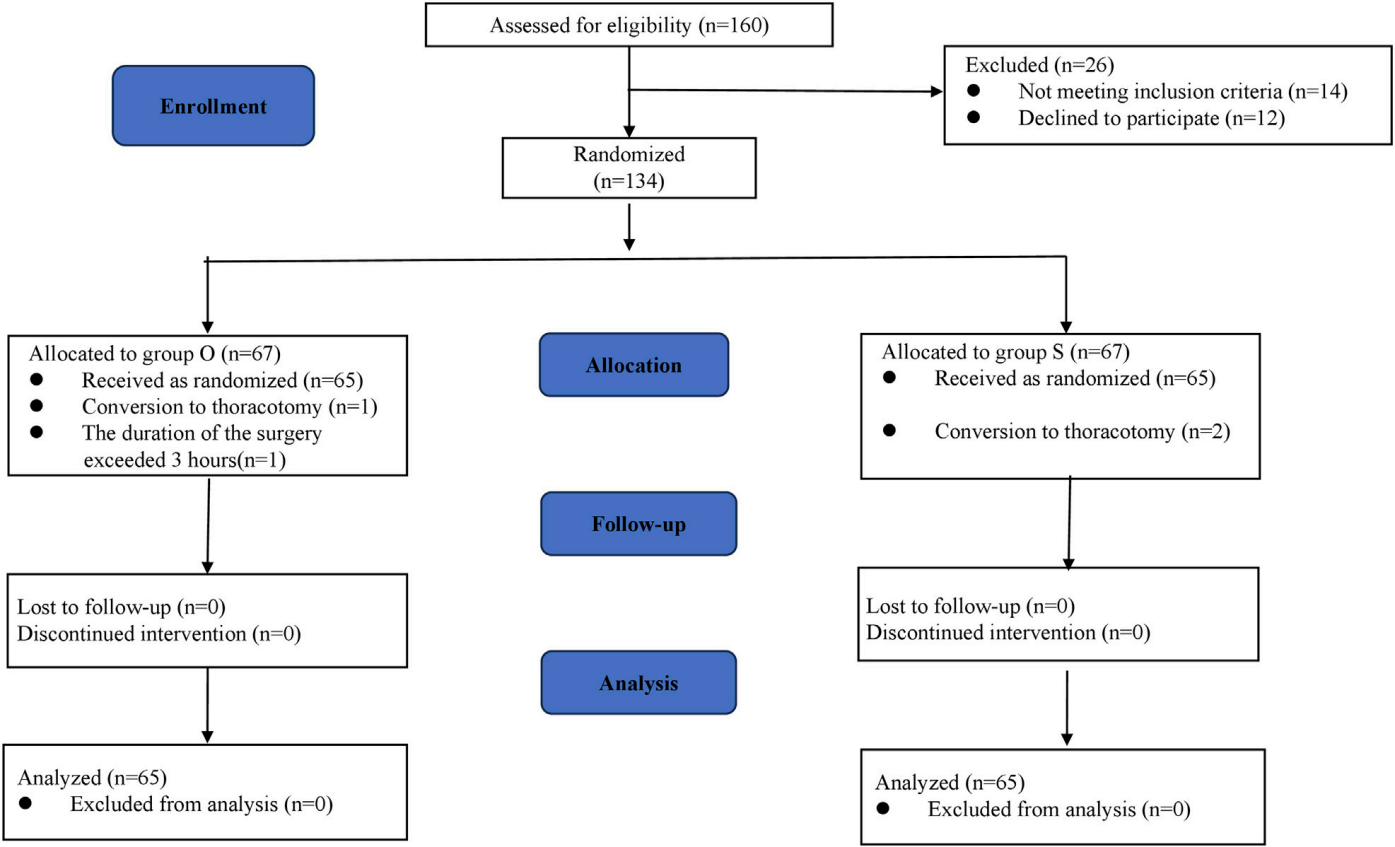
CONSORT flow chart.

A total of 160 patients were screened and 134 patients meet the criteria of this protocol from December 2024 to February 2025. Among them, 67 patients were randomly assigned to the oliceridine group and the sufentanil group. In the oliceridine group, 1 patient had massive hemorrhage and underwent thoracotomy, and another 1 patient had a surgery lasting more than 3 h. In the sufentanil group, 2 patients were converted to thoracotomy. Finally, 130 patients were included in the final treatment analysis. The flow chart of the participants is shown in Figure 1.

Baseline and intraoperative characteristics were balanced between groups, with no clinically relevant differences observed (Table 1). BMI, ASA, Apfel scores, and comorbidities of the patients in the two groups were well - balanced, and the types of surgeries were similar. All participants had little difference in operation time, anesthesia time, intraoperative input volume, blood loss, urine volume, intraoperative sufentanil dose and intraoperative remifentanil consumption.

**TABLE 1:** Baseline and peri-operative data of patients receiving Oliceridine PCIA and Sufentanil PCIA. Values are number (proportion) or median [interquartile range].

Variable	Oliceridine group (n = 65)	Sufentanil group (n = 65)
Age (yr)	56 [51–59.5]	57 [51–61]
Sex:female	39 (60)	36 (55)
Height (cm)	165 [158.5–170]	165 [160–170]
Weight (kg)	64 [56–70]	66 [56–74]
BMI (kg/m^2^)	23.3 [22.0–26.5]	24.4 [22.7–26.5]
ASAⅠ/Ⅱ/Ⅲ		
Ⅰ	3 (4.6)	2 (3.1)
Ⅱ	54 (83.1)	52 (80)
Ⅲ	8 (12.3)	11 (16.9)
Apfel PONV risk score		
1	2 (3.1)	2 (3.1)
2	24 (36.9)	26 (40)
3	39 (60)	37 (56.9)
Total risk score	3 (2–3)	3 (2,3)
Comorbidities		
Hypertension	14 (21.5)	16 (24.6)
Diabetes	4 (6.2)	6 (9.2)
Lacunar infarction	4 (6.2)	5 (7.7)
Length of surgery (min)	120 [80–150]	120 [85–150]
Length of anesthesia (min)	155 [120–180]	150 [120–182.5]
Intravenous fluids (mL)	1,110 [920–1,350]	1,020 [880–1,255]
Intraoperative bleeding (mL)	30 [20–47.5]	30 [25–50]
Urinary volume (ml)	530 [405–655]	550 [400–660]
Intraoperative sufentanil dose (μg)	32 [28–35]	33 [28–38]
Intraoperative remifentanil consumption (μg)	1800 [1,520–2,240]	1750 [1,470–2,180]
Sites of VATS		
Left	28 (43.1)	26 (40)
Right	37 (56.9)	39 (60)
Surgical procedure		
Wedge resection	20 (30.8)	19 (29.2)
Segmentectomy	25 (38.5)	22 (33.8)
Lobectomy	20 (30.8)	24 (36.9)

The intraoperative hemodynamic parameters, including mean arterial pressure (MAP) and heart rate (HR), were comparable between the two groups at all measured time points (baseline, induction, intubation, surgical incision (Table 2). There were no statistically significant differences in the incidence of hypotension (MAP <65 mmHg) or hypertension (MAP >110 mmHg) between the groups (all P > 0.05). The use of vasoactive agents (e.g., ephedrine, phenylephrine, or urapidil) was also similar between groups (P > 0.05).

**TABLE 2:** Intraoperative Hemodynamic Parameters and Vasoactive Drug Use. Values are mean ± standard deviation, number (proportion) as appropriate.

Parameter	Oliceridine group (n = 65)	Sufentanil group (n = 65)	P value
Mean Arterial Pressure (MAP), mmHg			
Baseline	85.3 ± 8.1	84.2 ± 9.6	0.712
Induction	78.0 ± 10.3	76.2 ± 11.6	0.453
Intubation	92.1 ± 12.4	94.5 ± 13.0	0.621
Surgical incision	88.0 ± 9.2	87.7 ± 10.2	0.789
Heart Rate (HR), beats/min			
Baseline	72.9 ± 11.0	74.0 ± 10.2	0.423
Induction	68.5 ± 9.6	67.8 ± 8.5	0.671
Intubation	84.4 ± 13.5	86.0 ± 12.4	0.554
Surgical incision	79.3 ± 10.0	81.1 ± 11.3	0.482
Hypotension (MAP <65 mmHg)	12 (18.5)	15 (23.1)	0.517
Hypertension (MAP >110 mmHg)	8 (12.3)	6 (9.2)	0.571
Vasoactive drug use			
Ephedrine	10 (15.4)	12 (18.5)	0.640
Phenylephrine	7 (10.8)	5 (7.7)	0.545
Urapidil	6 (9.2)	4 (6.2)	0.510

**FIGURE 2:**
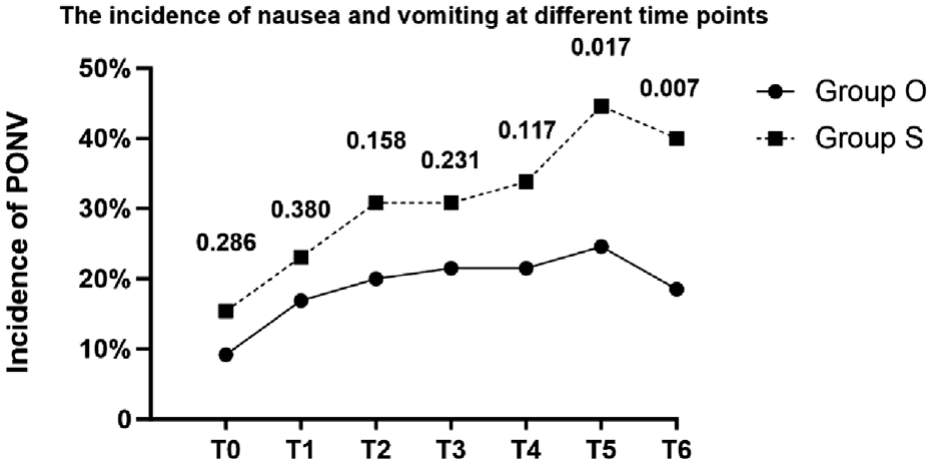
Line chart of the incidence of PONV. The numbers above the time points indicate P values (T0:Immediately after extubation; T1:30 min after extubation; T2:2 h after extubation; T3:6 h after extubation; T4:12 h after extubation; T5:24 h after extubation; T6:48 h after extubation).

The incidence of postoperative nausea and vomiting and the use of rescue anti - emetic drugs are shown in Table 3. The incidence of 48-h PONV, the primary endpoint, was 32.3% (21/65) in the oliceridine group versus 50.8% (33/65) in the sufentanil group (OR = 0.46; 95% CI, 0.23–0.94; P = 0.033). At each time point, the incidence of nausea and vomiting in the sufentanil group was higher than that in the oliceridine group at multiple time points, but not all time points had statistical significance. For example, at 24 h after surgery, the incidence of nausea/vomiting in the oliceridine group was 24.6% (16 cases), and that in the sufentanil group was 44.6% (29 cases) (P = 0.017). Regarding the demand for rescue anti - emetic drugs, the proportion in the sufentanil group was 23.1% (15 cases), which was higher than 13.8% (9 cases) in the oliceridine group, but the difference was not statistically significant (P = 0.175). In addition, moderate-to-severe PONV (VAS ≥4) occurred in 18.5% (12/65) of Group O versus 38.5% (25/65) of Group S (OR = 0.36; 95% CI, 0.16–0.81; P = 0.012). Figure 2 shows the incidence of PONV in the two groups at different time points.

**TABLE 3:** The incidence of postoperative nausea, vomiting, and use of rescue antiemetics.Values are number (proportion) or median [interquartile range].

Outcome	Oliceridine group (n = 65)	Sufentanil group (n = 65)	P value	Odds ratio or median difference (95% Cl)
Incidence of Nausea/vomiting				
Overall	21(32.3)/5(7.7)	33(50.8)/8(12.3)	0.033/0.380	0.46 [0.23–0.94] /0.59 [0.18–1.92]
Immediately after extubation	6 (9.2) /1 (1.5)	10(15.4)/0(00.0)	0.286/1.000	0.56 [0.19–1.64] /3.07 [0.12–74.5]
30 min after extubation	11(16.9)/0(00.0)	15(23.1)/2(3.1)	0.380/0.496	0.68 [0.29–1.62] /0.19 [0.01–4.06]
2 h after extubation	13(20.0)/2(3.1)	20(30.8)/3(4.6)	0.158/1.000	0.56 [0.25–1.26] /0.66 [0.11–4.06]
6 h after extubation	14(21.5)/2(3.1)	20(30.8)/3(4.6)	0.231/1.000	0.62 [0.28–1.36] /0.66 [0.11–4.06]
12 h after extubation	14(21.5)/3(4.6)	22(33.8)/5(7.7)	0.117/0.718	0.54 [0.25–1.17] /0.58 [0.13–2.54]
24 h after extubation	16(24.6)/5(7.7)	29(44.6)/8(12.3)	0.017/0.380	0.41 [0.19–0.86] /0.59 [0.18–1.92]
48 h after extubation	12(18.5)/3(4.6)	26(40.0)/7(10.7)	0.007/0.188	0.43 [0.15–0.76] /0.40 [0.09–1.62]
Need for rescue antiemetics	9 (13.8)	15 (23.1)	0.175	0.54 [0.22–1.33]
Moderate to severe nausea and vomiting (VAS≥4)	12 (18.5)	25 (38.5)	0.012	0.36 [0.16–0.81]

For key dichotomous outcomes (e.g., PONV, incidence), OR, with 95% CI, was calculated directly from 2 × 2 contingency tables to quantify effect sizes alongside χ^2^/Fisher’s exact tests.

**FIGURE 3:**
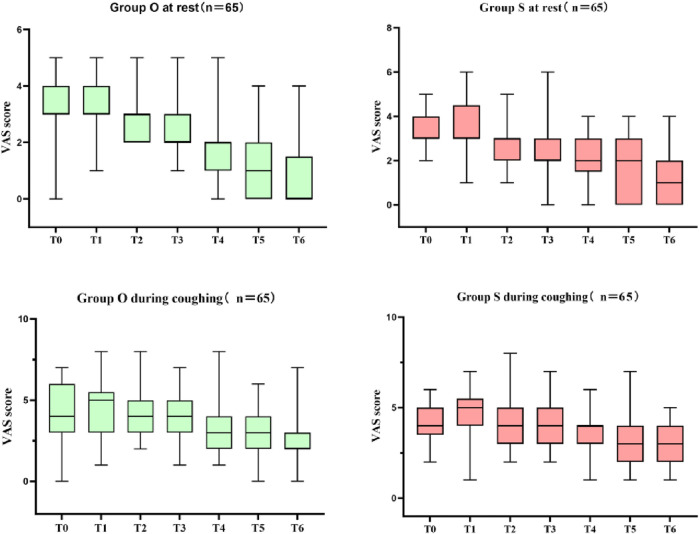
Box plot of VAS scores during rest and coughing (T0:Immediately after extubation; T1:30 min after extubation; T2:2 h after extubation; T3:6 h after extubation; T4:12 h after extubation; T5:24 h after extubation; T6:48 h after extubation).

**FIGURE 4:**
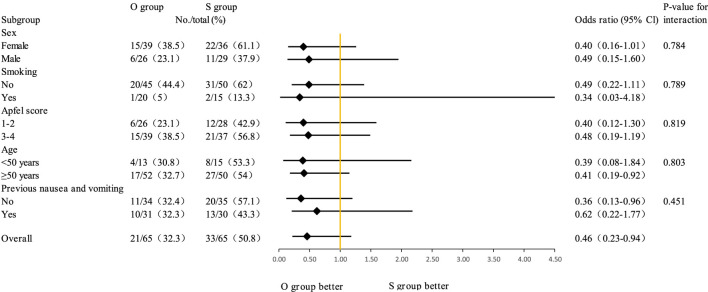
Subgroup analyses of PONV.

Table 4 presents the comparison of postoperative analgesia between the oliceridine group and the sufentanil group. In the early stage (Immediately after extubation, 30 min after extubation, 2 h after extubation, 6 h after extubation), there were no statistically significant differences in the scores between the two groups (all P - values were relatively large). Although statistically significant differences in VAS scores were observed at 12, 24, and 48 h (all P < 0.05), the absolute differences were small and likely not clinically relevant (e.g., median resting VAS of 0 vs. 1 at 48 h). Notably, there were no significant differences in rescue analgesic requirements between groups, supporting comparable clinical analgesic efficacy. The box plot of VAS scores during rest and coughing (Figure 3) shows that the VAS scores of the two groups fluctuated at different time points during rest and coughing, but no obvious score differences between the two groups were visually presented. Figure 4 is a subgroup analysis plot of PONV. The interaction P values for the gender subgroup, smoking status subgroup, Apfel score subgroup, age subgroup, and history of previous PONV were all large, indicating no significant interaction between subgroup factors and the association between the two groups.

**TABLE 4:** Postoperative analgesic profiles. Values are number (proportion) or median [interquartile range].

Variable	Oliceridine group(n =65)	Sufentanil group(n =65)	P value
VAS of pain (at rest/ during coughing)			
Immediately after extubation	3 [3,4]/4 [3–6]	3 [3,4]/4 [3.5-5]	0.339/0.819
30 min after extubation	3 [3,4]/5 [3-5.5]	3 [3–4.5]/5 [4–5.5]	0.154/0.300
2 h after extubation	3 [2,3]/4 [3–5]	3 [2,3]/4 [3–5]	0.156/0.530
6 h after extubation	2 [2,3]/4 [3–5]	2 [2,3]/4 [3–5]	0.693/0.187
12 h after extubation	2 [1,2]/3 [2–4]	2 [1.5-3]/4 [3,4]	0.165/0.019
24 h after extubation	1 [0–2]/3 [2–4]	2 [0–3]/3 [2–4]	0.071/0.035
48 h after extubation	0 [0–1.5]/2 [2,3]	1 [0–2]/3 [2–4]	0.042/0.023
Need for rescue analgesics			
0-24h	13(20)	17(26.2)	0.405
24-48h	10(15.4)	15(23.1)	0.266
Number of patients who clamped the IV-PCIA	26(40)	31(47.7)	0.377

Regarding the postoperative recovery of the two groups, there were no statistically significant differences in the time of first food intake, first flatus, first activity, and the Ramsay sedation score and BCS at 24 h and 48 h (Table 5). However, at 24 h, QoR-15 scores were significantly higher in Group O (median 112 [IQR 105–118]) than in Group S (median 107 [IQR 103–114]) (median difference = 5.0; 95% CI, 1.2–8.8; P = 0.024). This advantage persisted at 48 h (median 122 [113.5–128] vs. 116 [113–122]; median difference = 6.0; 95% CI, 2.1–9.9; P = 0.018). Group O reported lower insomnia scores at 24 h (median 13 (Food and Drug Administration, 2020; DeWire et al., 2013; Ni et al., 2024; Bergese et al., 2019; Biskupiak et al., 2024; An et al., 2021) vs. 14 [12.5–16]; median difference = −1.0; 95% CI, −2.3 to −0.5; P = 0.009) and 48 h (median 12 (Food and Drug Administration, 2020; DeWire et al., 2013; Ni et al., 2024; Bergese et al., 2019; Biskupiak et al., 2024) vs. 13 (Ni et al., 2024; Bergese et al., 2019; Biskupiak et al., 2024; An et al., 2021); median difference = −1.0; 95% CI, −2.0 to −0.5; P = 0.008).

**TABLE 5:** Postoperative recovery. Values are median [interquartile range].

Variable	Oliceridine group(n =65)	Sufentanil group(n =65)	P value
Time to first oral intake (hour)	13 [7.5–16]	11 [6–14]	0.111
Time to first flatus (hour)	25 [20–30]	24 [18–30]	0.965
Time to first mobilization (hour)	23 [11,20–23]	23 [11,20–23]	0.685
Recovery time (min)	30 [30–40]	30 [30–40]	0.668
Time to remove drainage tube (hour)	70 [48–91]	72 [47.5–90]	0.749
Hospital Discharge time (day)	4 [3.5–5]	4 [3.8–5.3]	0.745
Ramsay sedation score			
24 h	2 [2]	2 [2]	0.302
48 h	2 [2]	2 [2]	0.405
Bruggrmann comfort scale			
24 h	2 [1,2]	2 [1,2]	0.529
48 h	2 [2]	2 [2]	0.473
Quality of recovery-15			
24 h	112 [105–118]	107 [103–114]	0.024
48 h	122 [113.5–128]	116 [113–122]	0.018
Athens insomnia scale			
24 h	13 [10–15]	14 [12.5–16]	0.009
48 h	12 [10–14]	13 [12–15]	0.008

Table 6 compares the safety outcomes of the oliceridine group (n = 65) and the sufentanil group (n = 65). There were no statistically significant differences in the incidences of dizziness, nightmares or hallucinations between the two groups, and no cases of respiratory depression, dry mouth, allergy, or bradycardia occurred. The incidence of hypotension was 3.1% in the oliceridine group and 1.5% in the sufentanil group, with no statistically significant difference.

**TABLE 6:** Safety outcomes. Values are number (proportion).

Adverse event	Oliceridine group (n = 65)	Sufentanil group (n = 65)	P value
Safety outcomes			
Dizziness	13 (20)	15 (23.1)	0.670
Nightmare or hallucination	3 (4.6)	2 (3.1)	0.648
Respiratory depression	0	0	1.000
Dry mouth	0	0	1.000
Allergy	0	0	1.000
Bradycardia	0	0	1.000
Hypotension	2 (3.1)	1 (1.5)	1.000

## Discussion

### Contextualizing novelty

Our findings extend beyond confirming oliceridine’s established PONV reduction in three key aspects:

First RCT demonstrating oliceridine’s superiority over sufentanil in thoracoscopic surgery—a setting where opioid-induced PONV complicates >40% of cases (An et al., 2021) and impedes early recovery.

Significantly higher QoR-15 scores (+5.0 points at 24 h, P < 0.05) and improved sleep (AIS reduction −1.0 point, P = 0.009) suggest oliceridine enhances recovery beyond PONV reduction, a dimension underreported in prior literature.

With comparable rescue analgesia needs but superior PONV control, oliceridine provides a viable alternative to sufentanil in protocols where gastrointestinal tolerability limits opioid optimization.

### Superiority in reducing PONV

The primary finding was that oliceridine significantly reduced 48-h PONV incidence (32.3% vs. 50.8%, P = 0.033) and moderate-to-severe PONV (18.5% vs. 38.5%, P = 0.012) compared to sufentanil, with the greatest advantage observed within 24 h (24.6% vs. 44.6%, P = 0.017). This aligns with the pharmacological properties of oliceridine: its G protein bias minimizes β-arrestin-mediated gastrointestinal adverse effects (Tan and Habib, 2021a), consistent with APOLLO trials showing fewer gastrointestinal reactions versus morphine (Viscusi et al., 2019; Singla et al., 2019) and a meta-analysis reporting lower nausea/vomiting with oliceridine (Niu et al., 2023). Importantly, beyond reducing gastrointestinal motility inhibition (e.g., delayed gastric emptying), β-arrestin pathway activation directly stimulates 5-HT_3_ and D_2_ receptors in the area postrema of the brainstem, a core mechanism of PONV (DeWire et al., 2013). Oliceridine’s low potency for recruiting β-arrestin may reduce this central activation of emetic receptors, providing a receptor-level explanation for the observed reduction in PONV. The significant reduction in PONV with oliceridine holds particular value for high-risk subgroups, such as females (60% of cohort), non-smokers, and patients with Apfel scores ≥3 (58% of cohort)—populations where conventional opioids exacerbate emetogenic susceptibility. Notably, rescue antiemetic use did not differ significantly between groups (13.8% vs. 23.1%, P = 0.175), likely due to the small sample size (n = 65 per group). Larger studies, such as a meta-analysis including over 1,000 patients, demonstrated reduced rescue antiemetic needs with oliceridine (Beard et al., 2021), suggesting our non-significant result may reflect underpowering, warranting validation in larger cohorts.

### Analgesic efficacy and duration

Early postoperative pain scores (0–6 h) were comparable between groups, but sufentanil showed higher VAS scores at 12–48 h, indicating oliceridine’s superior long-term analgesic stability. Notably, this occurred despite using theoretically equianalgesic doses of both agents, suggesting oliceridine’s clinical advantage in sustained pain control. Sufentanil’s high lipophilicity and tendency for tissue sequestration may contribute to later analgesic fluctuations. In contrast, oliceridine’s shorter context-sensitive half-time (t_1_/_2_ ˜ 1.5–3 h) and linear pharmacokinetic profile likely enable more stable plasma concentrations during the PCIA maintenance phase, mitigating the risk of breakthrough pain (Markham, 2020). While the dose conversion was based on an equianalgesic model derived from prior APOLLO trials (oliceridine:morphine ≈1:5; sufentanil: morphine ≈1:1,000), the comparable early analgesia and potentially superior later analgesia observed here support the appropriateness of the chosen PCIA regimen. Future studies utilizing concentration-effect curves could validate the precision of this conversion specifically for PCIA delivery. Furthermore, oliceridine is metabolized by CYP2D6 and CYP3A4 with predictable clearance, and no dose adjustment is needed for mild-to-moderate hepatic/renal impairment (Tan and Habib, 2021b). This metabolic stability likely contributes to consistent analgesic effects without unexpected accumulation or rapid decline, supporting sustained efficacy throughout the 48-h PCIA period. Despite these differences, rescue analgesic requirements were similar, confirming both drugs meet clinical analgesic needs.

### Improved recovery and sleep quality

Oliceridine significantly increased Quality of Recovery-15 (QoR-15) scores and reduced Athens Insomnia Scale (AIS) scores at 24–48 h, reflecting better overall recovery and sleep. This may result from: (1) reduced PONV, avoiding electrolyte imbalance and discomfort; (2) sustained analgesia after 12 h, minimizing pain-related sleep disruption; and (3) fewer opioid-related adverse events (ORAE), aligning with findings that oliceridine enhances patient-reported outcomes versus traditional opioids (Soergel et al., 2014; Nafziger et al., 2020). Although these differences were statistically significant, their clinical relevance remains to be fully established.

### Safety profile

No significant differences in adverse events (dizziness, hypotension) were observed, with no cases of respiratory depression, allergy, or bradycardia in either group. These results are encouraging but should be interpreted cautiously given the small sample size. Larger trials (e.g., APOLLO-1 and APOLLO-2) have confirmed oliceridine’s favorable safety profile, with lower rates of respiratory and gastrointestinal adverse events versus morphine (Singla et al., 2019; Tan and Habib, 2021a), supporting its safety in clinical use.

#### Subgroup consistency

Subgroup analyses (gender, smoking status, Apfel score, age, and prior PONV history) showed no significant interaction with treatment effects, confirming oliceridine’s consistent PONV-reducing efficacy across diverse populations. This stability strengthens its clinical applicability.

### Integration with ERAS goals

While ERAS pathways emphasize opioid-sparing strategies to minimize adverse effects like PONV, managing moderate-to-severe pain often necessitates potent opioid intervention. Our findings position oliceridine as a promising solution to this core ERAS dilemma: it provided comparable analgesic efficacy to sufentanil (evidenced by similar rescue analgesia rates), while simultaneously delivering significant reductions in PONV—a key ERAS barrier. Furthermore, oliceridine uniquely enhanced patient-reported recovery (QoR-15 + 5.0 points at 24 h) and improved sleep quality (AIS reduction −1.0 point), aligning with ERAS objectives beyond analgesia (Gelman et al., 2018; Echeverria-Villalobos et al., 2020). This dual advantage—effective pain control coupled with improved tolerability and recovery metrics—supports oliceridine’s role in protocols where gastrointestinal side effects limit opioid optimization.

This study has several limitations. First, the oliceridine dose was selected based on estimated morphine equivalence and pharmacological data, but the precise potency ratio relative to sufentanil and the optimal PCIA dose for oliceridine warrant further investigation in larger, dose-finding studies, considering potential influences of patient factors. Second, the evaluation period was limited to 48 h; longer-term outcomes were not assessed. Although this period covers the peak incidence of PONV, it does not assess the potential long-term impact of pain and PONV on functional recovery. Notably, PONV is known to delay mobilization, and delayed mobilization itself is associated with an increased risk of chronic pain. Extending follow-up could reveal potential long-term benefits of oliceridine on functional outcomes, such as reduced chronic pain incidence. Third, while safety outcomes were encouraging and aligned with prior literature, the sample size was insufficient to robustly compare rates of less common adverse events. Fourth, the study was conducted at a single center.

## Conclusion

In thoracoscopic surgery patients, oliceridine-based PCIA was associated with a significantly lower incidence of PONV and modest improvements in recovery quality and sleep scores compared to sufentanil, though the clinical impact of these differences requires further study. These findings suggest oliceridine is a viable alternative for postoperative analgesia, warranting further validation in larger cohorts.

## Data Availability

The raw data supporting the conclusions of this article will be made available by the authors, without undue reservation.

## References

[B1] An G. Zhang Y. Chen N. Fu J. Zhao B. Zhao X. (2021). Opioid-free anesthesia compared to opioid anesthesia for lung cancer patients undergoing video-assisted thoracoscopic surgery: a randomized controlled study. PLoS One 16 (9), e0257279. 10.1371/journal.pone.0257279 34555043 PMC8460000

[B2] Apfel C. C. Läärä E. Koivuranta M. Greim C. A. Roewer N. (1999). A simplified risk score for predicting postoperative nausea and vomiting: conclusions from cross-validations between two centers. Anesthesiology 91 (3), 693–700. 10.1097/00000542-199909000-00022 10485781

[B3] Apfel C. C. Heidrich F. M. Jukar-Rao S. Jalota L. Hornuss C. Whelan R. P. (2012). Evidence-based analysis of risk factors for postoperative nausea and vomiting. Br. J. Anaesth. 109 (6), 742–753. 10.1093/bja/aes276 23035051

[B4] Apfelbaum J. L. Chen C. Mehta S. S. Gan T. J. (2003). Postoperative pain experience: results from a national survey suggest postoperative pain continues to be undermanaged. Anesth. Analg. 97 (2), 534–540. 10.1213/01.ANE.0000068822.10113.9E 12873949

[B5] Beard T. L. Michalsky C. Candiotti K. A. Viscusi E. R. Singla N. K. Soergel D. G. (2021). Oliceridine is associated with reduced risk of vomiting and need for rescue antiemetics compared to morphine: exploratory analysis from two phase 3 randomized placebo and active controlled trials. Pain Ther. 10 (1), 401–413. 10.1007/s40122-020-00216-x 33210266 PMC8119517

[B6] Bergese S. D. Brzezinski M. Hammer G. B. Beard T. L. Pan P. H. Mace S. E. (2019). ATHENA: a phase 3, open-label study of the safety and effectiveness of oliceridine (TRV130), A G-Protein selective agonist at the µ-Opioid receptor, in patients with moderate to severe acute pain requiring parenteral opioid therapy. J. Pain Res. 12, 3113–3126. 10.2147/JPR.S217563 31814753 PMC6861532

[B7] Biskupiak J. Oderda G. Brixner D. Wandstrat T. L. (2024). Gastrointestinal adverse effects associated with the use of intravenous oliceridine compared with intravenous hydromorphone or fentanyl in acute pain management utilizing adjusted indirect treatment comparison methods. J. Comp. Eff. Res. 13 (5), e230041. 10.57264/cer-2023-0041 38497192 PMC11036942

[B8] DeWire S. M. Yamashita D. S. Rominger D. H. Liu G. Cowan C. L. Graczyk T. M. (2013). A G protein-biased ligand at the μ-opioid receptor is potently analgesic with reduced gastrointestinal and respiratory dysfunction compared with morphine. J. Pharmacol. Exp. Ther. 344 (3), 708–717. 10.1124/jpet.112.201616 23300227

[B9] Echeverria-Villalobos M. Stoicea N. Todeschini A. B. Arias-Morales C. E. Gonzalez-Zacarias A. A. Fabara S. P. (2020). Enhanced recovery after surgery (ERAS): a perspective review of postoperative pain management under eras pathways and its role on opioid crisis in the United States. Clin. J. Pain 36 (3), 219–226. 10.1097/AJP.0000000000000792 31868759

[B10] Food and Drug Administration (2020). FDA approves new opioid for intravenous use in hospitals, other controlled clinical settings. Available online at: https://www.fda.gov/news-events/press-announcements/fda-approves-new-opioid-intravenous-use-hospitals-other-controlled-clinical-settings.

[B11] Gelman D. Gelmanas A. Urbanaitė D. Tamošiūnas R. Sadauskas S. Maleckas A. (2018). Role of multimodal analgesia in the evolving enhanced recovery after surgery pathways. Med. Kaunas. 54 (2), 20. 10.3390/medicina54020020 30344251 PMC6037254

[B12] Ho K. Y. Gan T. J. (2006). Pharmacology, pharmacogenetics, and clinical efficacy of 5-hydroxytryptamine type 3 receptor antagonists for postoperative nausea and vomiting. Curr. Opin. Anaesthesiol. 19 (6), 606–611. 10.1097/01.aco.0000247340.61815.38 17093363

[B13] Markham A. (2020). Oliceridine: first approval. Drugs 80 (16), 1739–1744. 10.1007/s40265-020-01414-9 33025536

[B14] Nafziger A. N. Arscott K. A. Cochrane K. Skobieranda F. Burt D. A. Fossler M. J. (2020). The influence of renal or hepatic impairment on the pharmacokinetics, safety, and tolerability of oliceridine. Clin. Pharmacol. Drug Dev. 9 (5), 639–650. 10.1002/cpdd.750 31697049 PMC7383509

[B15] Ni Y. Huang R. Yang S. Wu Y. Liu J. Xie Z. (2024). Pharmacokinetics and safety of oliceridine fumarate injection in Chinese patients with chronic non-cancer pain: a phase I, single-ascending-dose, open-label clinical trial. Drug Des. Devel Ther. 18, 2729–2743. 10.2147/DDDT.S461416 38974123 PMC11227858

[B16] Niu J. Hu W. Lu Y. Tang H. (2023). Efficacy and safety of oliceridine treatment in patients with postoperative pain: a systematic review and meta-analysis of randomized controlled trials. Expert Rev. Clin. Pharmacol. 16 (6), 589–599. 10.1080/17512433.2023.2213889 37171148

[B17] Öbrink E. Jildenstål P. Oddby E. Jakobsson J. G. (2015). Post-operative nausea and vomiting: update on predicting the probability and ways to minimize its occurrence, with focus on ambulatory surgery. Int. J. Surg. 15, 100–106. 10.1016/j.ijsu.2015.01.024 25638733

[B18] Paul A. K. Smith C. G. Rahmattulla M. Sarker M. M. R. Hossen M. S. Jamaluddin M. F. (2021). Opioid analgesia and opioid-induced adverse effects: a review. Pharmaceutics 14 (11), 1091. 10.3390/ph14111091 PMC8620360 34832873

[B19] Shafi S. Collinsworth A. W. Copeland L. A. Ogola G. O. Qiu T. Kouznetsova M. (2018). Association of opioid-related adverse drug events with clinical and cost outcomes among surgical patients in a large integrated health care delivery system. JAMA Surg. 153 (8), 757–763. 10.1001/jamasurg.2018.1039 29799927 PMC6142954

[B20] Singla N. K. Skobieranda F. Soergel D. G. Salamea M. Burt D. A. Demitrack M. A. (2019). APOLLO-2: a randomized, placebo and active-controlled phase III study investigating oliceridine (TRV130), a G protein-biased ligand at the μ-Opioid receptor, for management of moderate to severe acute pain following abdominoplasty. Pain Pract. 19 (7), 715–731. 10.1111/papr.12801 31162798 PMC6851842

[B21] Soergel D. G. Subach R. A. Sadler B. Connell J. Marion A. S. Cowan C. L. (2014). First clinical experience with TRV130: pharmacokinetics and pharmacodynamics in healthy volunteers. J. Clin. Pharmacol. 54 (3), 351–357. 10.1002/jcph.207 24122908

[B22] Sun Q. Li Z. Wang Z. Liu X. Wang C. Pan H. (2023). Immunosuppression by opioids: mechanisms of action on innate and adaptive immunity. Biochem. Pharmacol. 209, 115417. 10.1016/j.bcp.2023.115417 36682388

[B23] Tan H. S. Habib A. S. (2021a). Oliceridine: a novel drug for the management of moderate to severe acute pain - a review of current evidence. J. Pain Res. 14, 969–979. 10.2147/JPR.S278279 33889018 PMC8054572

[B24] Tan H. S. Habib A. S. (2021b). Safety evaluation of oliceridine for the management of postoperative moderate-to-severe acute pain. Expert Opin. Drug Saf. 20 (11), 1291–1298. 10.1080/14740338.2021.1965989 34370562

[B25] Viscusi E. R. Skobieranda F. Soergel D. G. Cook E. Burt D. A. Singla N. (2019). APOLLO-1: a randomized placebo and active-controlled phase III study investigating oliceridine (TRV130), a G protein-biased ligand at the µ-opioid receptor, for management of moderate-to-severe acute pain following bunionectomy. J. Pain Res. 12, 927–943. 10.2147/JPR.S171013 30881102 PMC6417853

[B26] Wang H. Liu X. Wang T. Qin X. Sun C. Yu Y. (2017). Postoperative pain experiences in Chinese adult patients after thoracotomy and video-assisted thoracic surgery. J. Clin. Nurs. 26 (15-16), 2744–2754. 10.1111/jocn.13789 28252817

[B27] Wang J. Zheng K. Wen Q. Sun J. (2023). Sufentanil combined with nalmefene reduce the adverse events in recovery period of patients undergoing uvulopalatopharyngoplasty - a randomized controlled trial. Heliyon 9 (2), e13241. 10.1016/j.heliyon.2023.e13241 36755590 PMC9900260

