# Supplementary material for: Comparison of oliceridine and sufentanil in patient - controlled intravenous analgesia for post - thoracoscopic nausea and vomiting: a prospective, double - blind, randomized controlled trial
Source: Front Pharmacol. 2025 Sep 26;16:1576154. doi: 10.3389/fphar.2025.1576154 (PMC12511045; doi:10.3389/fphar.2025.1576154)
Supplement: Supplementary file 1 [file Table1.docx]

### Ramsay Sedation Scale

| **Score** | **Clinical State** | **Description** |
| --- | --- | --- |
| **1** | **Anxious, agitated, or restless** | Patient anxious or agitated; may require intervention |
| **2** | **Cooperative, oriented, and tranquil** | Patient calm, follows commands, awake/alert (**Ideal postoperative state**) |
| **3** | **Responsive to commands only** | Patient drowsy but responds appropriately to verbal commands |
| **4** | **Asleep with brisk response to light glabellar tap or loud auditory stimulus** | Patient asleep; rapid response to physical/auditory stimulus |
| **5** | **Asleep with sluggish response to light glabellar tap or loud auditory stimulus** | Patient asleep; delayed response to stimulus |
| **6** | **Asleep with no response to stimulus** | Unarousable; no response to vigorous stimulus |

### Bruggrmann comfort scale（BCS）

| Score | Description |
| --- | --- |
| 0 | Continuous pain |
| 1 | No pain at rest, severe pain on deep - breathing or coughing |
| 2 | No pain when lying quietly, mild pain on deep - breathing or coughing |
| 3 | No pain on deep - breathing |
| 4 | No pain on coughing |

### ****Quality of Recovery-15 (QoR-15) Scale****

| **Domain** | **Item** | **Scale (0–10)** |
| --- | --- | --- |
| **Physical Comfort** | 1. Able to breathe easily | 0 (Poor) ↔ 10 (Excellent) |
|  | 2. Feeling rested | 0 (Poor) ↔ 10 (Excellent) |
|  | 3. Able to enjoy food | 0 (Poor) ↔ 10 (Excellent) |
|  | 4. Feeling rested | 0 (Never) ↔ 10 (Always) |
|  | 5. Able to pass urine normally | 0 (Severe Problem) ↔ 10 (No Problem) |
| **Emotional State** | 6. Feeling in control of self | 0 (Never) ↔ 10 (Always) |
|  | 7. Feeling anxious or frightened | **Reverse-scored:** 0 (Always) ↔ 10 (Never) |
|  | 8. Feeling depressed | **Reverse-scored:** 0 (Always) ↔ 10 (Never) |
|  | 9. Having general feelings of well-being | 0 (Poor) ↔ 10 (Excellent) |
| **Psychological Support** | 10. Getting support from hospital doctors/nurses | 0 (Poor) ↔ 10 (Excellent) |
|  | 11. Able to communicate with hospital staff | 0 (Poor) ↔ 10 (Excellent) |
| **Physical Independence** | 12. Able to look after personal toilet/washing unaided | 0 (Unable) ↔ 10 (Fully Able) |
|  | 13. Able to return to work or usual home activities | 0 (Unable) ↔ 10 (Fully Able) |
| **Pain** | 14. Moderate pain | **Reverse-scored:** 0 (Always) ↔ 10 (Never) |
|  | 15. Severe pain | **Reverse-scored:** 0 (Always) ↔ 10 (Never) |

**Athens Insomnia Scale (AIS)**

| **Item** | **Question** | **Score (0–3)** |
| --- | --- | --- |
| **1** | Sleep induction (time to fall asleep) | 0: **No problem** 1: **Slightly delayed** 2: **Markedly delayed** 3: **Very delayed/did not sleep** |
| **2** | Awakenings during the night | 0: **No problem** 1: **Minor problem** 2: **Considerable problem** 3: **Serious problem/did not sleep** |
| **3** | Final awakening earlier than desired | 0: **Not earlier** 1: **A little earlier** 2: **Markedly earlier** 3: **Much earlier/did not sleep** |
| **4** | Total sleep duration | 0: **Sufficient** 1: **Slightly insufficient** 2: **Markedly insufficient** 3: **Very insufficient** |
| **5** | Overall quality of sleep | 0: **Satisfactory** 1: **Slightly unsatisfactory** 2: **Markedly unsatisfactory** 3: **Very unsatisfactory** |
| **6** | Well-being during the day | 0: **Normal** 1: **Slightly decreased** 2: **Markedly decreased** 3: **Very decreased** |
| **7** | Functioning (physical/mental) during the day | 0: **Normal** 1: **Slightly decreased** 2: **Markedly decreased** 3: **Very decreased** |
| **8** | Sleepiness during the day | 0: **None** 1: **Mild** 2: **Considerable** 3: **Excessive** |
